# Tumor‐infiltrating immune cell score as an independent prognostic predictor for endometrial carcinoma: Insights from a comprehensive analysis of the immune landscape

**DOI:** 10.1002/cnr2.1939

**Published:** 2023-11-28

**Authors:** Liping Zhang, Qiaoying Zhu, Qi Zhao, Xueping Lin, Hui Song, Hong Liu, Guiquan Zhu, Shun Lu, Bangrong Cao

**Affiliations:** ^1^ Department of Clinical Laboratory, Sichuan Provincial Maternity and Child Health Care Hospital, Affiliated Women's and Children's Hospital of Chengdu Medical College Chengdu Medical College Chengdu China; ^2^ Department of Biobank, Sichuan Clinical Research Center for Cancer, Sichuan Cancer Hospital & Institute, Sichuan Cancer Center University of Electronic Science and Technology of China Chengdu China; ^3^ Department of Gynecologic Oncology, Sichuan Clinical Research Center for Cancer, Sichuan Cancer Hospital & Institute, Sichuan Cancer Center University of Electronic Science and Technology of China Chengdu China; ^4^ State Key Laboratory of Oral Diseases, National Clinical Research Centre for Oral Diseases, Department of Head and Neck Oncology, West China Hospital of Stomatology Sichuan University Chengdu China; ^5^ Radiation Oncology Key Laboratory of Sichuan Province, Sichuan Clinical Research Center for Cancer, Sichuan Cancer Hospital & Institute, Sichuan Cancer Center University of Electronic Science and Technology of China Chengdu China

**Keywords:** CIBERSORTx, endometrial cancer, immune cell infiltration, prognostic score

## Abstract

**Background:**

Immune cells are crucial components in the tumor microenvironment and have a significant impact on the outcomes of patients.

**Aims:**

Here, we aimed to establish a prognostic score based on different types of tumor‐infiltrating immune cells for Endometrial Carcinoma (EC).

**Methods and Results:**

We enrolled and analyzed 516 EC patients from The Cancer Genome Atlas. The relative abundance of 22 immune cells were estimated by using the CIBERSORTx algorithm. Cox regression was performed to identify potential prognostic immune cells, which were used to develop a Tumor‐infiltrating Immune Cell Score (TICS). The prognostic and incremental value of TICS for overall survival were compared with traditional prognostic factors using the C‐index and decision curves. Clustering analysis using all immune cells identified three immune landscape subtypes, which had weak correlation with survival. A TICS was constructed using CD8T cells, resting memory CD4 T cells, activated NK and activated DCs, and classified patients as low‐, moderate‐ and high‐risk subgroups. The low‐risk subgroup had higher tumor mutation burden and activation of IL2/STAT5, IL2/STAT3 and IFN‐gamma response pathways. Conversely, the high‐risk subgroup was associated with DNA copy number variation, hypoxia and EMT process. The TICS subgroups significantly predicted overall survival, which was independent of patient age, tumor stage, grade and molecular classification. Moreover, we developed a nomogram incorporating TICS and clinicopathologic factors, which significantly improved the predictive accuracy compared to the clinicopathologic model alone.

**Conclusion:**

The TICS is an effective and independent prognostic predictor for EC patients and may serve as a useful supplement to clinicopathological factors and molecular subtyping.

## 1 INTRODUCTION

Endometrial carcinoma (EC) is a common gynecologic malignancy, and its incidence rate ranks the sixth in female malignant tumors in the world.^1^ In 2020, it was estimated that about 417 367 peoples were diagnosed with EC and 97 370 cases died of this disease worldwide. Notably, the incidence of EC continues to rise especially in developed countries.^2^ According to pathogenetic classification, ECs are typically classified as type I (endometrioid adenocarcinomas) and type II (serous, clear cell and mixed cell carcinoma).^3^ Most patients are diagnosed with type I tumors, generally have a favorable prognosis. In contrast, type II tumors tend to be more clinically aggressive and associated with unfavorable outcomes. Recently, molecular classification based on integrated genomic analysis identified four distinct subgroups of ECs.^4^, ^5^ However, the current risk assessment system based on clinicopathologic factors or molecular classification are insufficient to predict outcomes accurately for some patients. Therefore, it is necessary to establish a novel prognostic index as a complementary parameter to improve the prognosis for patients with ECs.

The quantity and status of tumor‐infiltrating immune cells have been shown to impact patient outcomes in various cancers, including EC. Elevated levels of intra‐tumoral CD3+ tumor‐infiltrating lymphocytes (TILs), CD8+ T‐lymphocytes, NK cells, helper T cells and CD45R0+ memory T‐lymphocytes were associated with favorable disease‐free survival (DFS) and/or overall survival (OS) in EC patients.^6^, ^7^, ^8^, ^9^, ^10^, ^11^ Conversely, higher counts of FOXP3+ regulatory T‐cells and CD68+ macrophages have been linked to an immune‐exclusion phenotype and tumor recurrence.^7^, ^12^, ^13^ A recent study found that the expression of PD‐L1 on immune cells was significantly correlated with patient survival, highlighting the importance of immune checkpoints in prognosis.^14^ Talhouk et al. identified two immune clusters in EC tumors based on TIL patterns (including CD8+ T cells, CD4+ T cells, Tregs, B cells, and plasma cells) and the PD‐1/PD‐L1 pathway in both the epithelial and stromal compartments.^15^ However, this immune classification was not associated with prognosis of EC patients.^15^ These results indicate that the immune microenvironment of EC is complex, and it is necessary to systematically analyze the immune landscape and select valuable immune cells to establish a robust prognostic model.

CIBERSORTx (Cell‐type Identification By Estimating Relative Subsets Of RNA Transcripts, the next version) is a novel algorithm used to estimate the proportion of distinct cell types based on bulk gene expression data generated from RNA‐seq or microarray analyses.^16^ This algorithm has been demonstrated to outperform other methods in estimating the relative proportion of tumor‐infiltrating immune cells without physical cell sorting.^17^

In this study, we aimed to comprehensively analyze the prognostic value of different immune cells in tumor microenvironment and develop a robust predictive model based on immune cells to improve the accuracy of prognosis for EC. The CIBERSORTx was employed to evaluate the relative abundance of 22 immune cells in 516 EC tumors from The Cancer Genome Atlas (TCGA). We identified CD8+ T cells, resting memory CD4+ T cells, activated NK cells and activated DCs as essential prognostic indicators, which were combined to develop a Tumor‐infiltrating Immune Cell Score (TICS). The TICS could predict OS and RFS independently of age, tumor stage, pathology type and molecular classification. Meanwhile, the combination of TICS and clinicopathologic factors significantly improved the accuracy of prognosis. These results suggested that the TICS may serve as a useful supplement to existing factors such as pathology and molecular subtyping in predicting outcome of EC patients.

## 2 MATERIALS AND METHODS

### 2.1 Patients and gene expression data

The cohort included in this study consisted of 545 cases of Uterine Corpus Endometrial Carcinoma (UCEC) obtained from The Cancer Genome Atlas (TCGA) portal (https://portal.gdc.cancer.gov/repository). Demographic and clinicopathological characteristics of the patients, as well as gene expression profiles were downloaded from the TCGA portal at April 24, 2022. The inclusion criteria for patient selection were: (1) diagnosed with uterine corpus endometrial cancer by pathology, (2) without chemotherapy or radiotherapy before sampling, (3) with follow‐up information, and (4) with RNA‐sequence data of the primary tumor. Moreover, patients with tumors failed in the CIBERSORTx analysis (as defined bellow) were excluded. Finally, a total of 516 EC patients were included in the study. Overall survival (OS) and progression‐free survival (PFS) information were used for prognosis analysis.

For gene expression data, the raw counts of RNA‐seq data were downloaded from TCGA Data Portal. Data normalization across samples was performed by using the transcripts per million (TPM) method. Gene annotation was performed with the Ensembl database (http://ensembl.org/biomart/martview/). The gene expression value was log2‐transformed for subsequent analysis.

### 2.2 Estimation of immune cell fractions

The relative fractions of 22 subtypes of immune cells in each tumor tissue were estimated by using the CIBERSORTx.^16^ Normalized RNA‐seq data (TPM) was uploaded to the CIBERSORTx Web site (https://cibersortx.stanford.edu/). Running parameters included: (1) using the LM22 signature matrix, (2) selecting B‐mode for batch correction, and (3) setting 1000 permutations for significance analysis. Tumor samples with *p*‐value >.05 were considered to be failed in the deconvolution analysis and were excluded for further analysis.

### 2.3 Construction of tumor‐infiltrating immune cell score (TICS)

The predictive capabilities for survival of tumor infiltrating immune cell fractions were evaluated by Cox regression. Multivariable analysis was performed by adding the traditional prognostic factors to estimate the independent prognostic capacities of immune cells. Variables with *p*‐value <.05 in multivariable Cox regression were used to construct a prognostic immune score. For each candidate prognostic immune cell, the optimal cutoff point was determined by the maximally selected rank statistics,^18^, ^19^ which were calculated by using the R software “survminer.” Patients were classified as two groups by the cutoff point of each immune cell, and received a point of 0 and 1 for low‐ and high‐risk, respectively. The prognostic immune score was defined as the sum of risk scores of all candidate immune cells.

### 2.4 Genetic and biological characteristics of TICS

The genetic alterations of the TCGA samples such as tumor mutation burden (TMB), number of DNA segments and aneuploidy score were obtained from previously published data.^20^ The predicted tumor neoantigen counts for each sample were also downloaded. T‐cell repertoire (TCR) and B‐cell repertoire (BCR) diversities were obtained from previous study.^20^ The four molecular subtypes of EC (POLE mutation, MSI‐high, TP53‐abnormal and NSMP for none specific molecular profile) was defined as previously described.^4^, ^21^

The relationship of TICS subgroups with TNM stage and molecular subtypes were visualized by an alluvial diagram. Gene Set Enrichment Analysis (GSEA) was performed through the GSEA software (http://www.broadinstitute.org/gsea/). The biological meanings of low‐, middle‐ and high‐risk subgroups according to TICS were studied by using the 50 hallmark gene sets from the MSigDB.^22^ Bonferroni correction was used for multiple testing. Ridgeline plots were used to visualize the significant gene sets. The significance of differences in TMB, neoantigen counts, DNA segments, aneuploidy score, TCR diversity and BCR diversity were estimated by using the Mann–Whitney test or Kruskal–Wallis rank sum test.

### 2.5 Statistical analysis

Statistical differences in categorical variables among different groups were calculated using the Chi‐square test or Fisher's exact test. The *t*‐test or Mann–Whitney test were used for continuous variables. Unsupervised hierarchical clustering analysis was used to identify the overall immune subtypes of ECs based on the fractions of 22 immune cells. Spearman correlation was used to calculate the correlation of the infiltrating fractions of different immune cells, and this was visualized using the R package “corrplot.” The correlation between CD8+ T cells and resting memory CD4+ T cells was presented by a scatter plot.

Multivariable Cox regression analysis was performed using TICS and other clinicopathologic features, including age, menopause status, tumor stage, tumor grade, pathological subtype, and molecular subtype. A back and forward stepwise variable selection procedure was performed using the Akaike information criterion (AIC). The last Cox model was used to create a nomogram predictor. The accuracy and discrimination of predictions were measured by calibration curves, decision curve and C‐index. The results of C‐index were calculated by bootstrap with 1000 times of resample. Kaplan–Meier curves and log‐rank test were used to compare the OS or PFS of different patient subgroups. All statistical tests were two‐sided, and a *p*‐value <.05 was considered significant. R software (v4.0.5) was used for all statistical analyses.

## 3 RESULTS

### 3.1 Overall intratumoral immune landscapes of endometrial cancer

A total of 516 patients from the TCGA cohort were involved in this study. Clinical characteristics are listed in Table 1. The median age of all patients was 64 years old (interquartile range, from 57 to 71 years). The majority of patients were post‐menopause (81.8%) with tumors of endometrioid histology (73.6%), grade 3 (59.5%), and TNM stage I (62.2%).

**TABLE 1:** Comparison of TICS groups with clinicopathological variables.

		TICS groups (number and percentage)			
Variables	Total (*N* = 516)	Low risk	Moderate risk	High risk	*p*‐Value
Age					.504
<70 years	362 (70.2)	252 (71.6)	73 (65.8)	37 (69.8)	
≥70 years	154 (29.8)	100 (28.4)	38 (34.2)	16 (30.2)	
Age* (years)	64 (57–71)	63 (56.25–71)	65 (57.5–73)	66 (61–72)	.114
Height* (cm)	161 (157–166)	161 (157–166)	160.5 (156–167)	163 (157–165.5)	.96
Weight* (kg)	84 (67–102.5)	84 (66–102)	85.5 (72–113)	76 (62.5–94)	.117
Menopause_status					.771
Pre	33 (6.4)	21 (6.0)	10 (9.0)	2 (3.8)	
Post	422 (81.8)	286 (81.2)	89 (80.2)	47 (88.7)	
Indeterminate	16 (3.1)	13 (3.7)	3 (2.7)	0 (0)	
Peri	17 (3.3)	13 (3.7)	2 (1.8)	2 (3.8)	
N/A	28 (5.4)	19 (5.4)	7 (6.3)	2 (3.8)	
Histology type					.137
Endometrioid	380 (73.6)	263 (74.7)	84 (75.7)	33 (62.3)	
Serous or mixed	136 (26.4)	89 (25.3)	27 (24.3)	20 (37.7)	
Tumor grade					.084
G1	95 (18.4)	76 (21.6)	14 (12.6)	5 (9.4)	
G2	114 (22.1)	76 (21.6)	27 (24.3)	11 (20.8)	
G3	307 (59.5)	200 (56.8)	70 (63.1)	37 (69.8)	
Molecular type					<.001
MSI	153 (29.7)	121 (34.4)	22 (19.8)	10 (18.9)	
NSMP	169 (32.8)	109 (31.0)	46 (41.4)	14 (26.4)	
POLE	41 (7.9)	36 (10.2)	5 (4.5)	0 (0)	
TP53	153 (29.6)	86 (24.4)	38 (34.2)	29 (54.7)	
Tumor stage					.008
I	321 (62.2)	234 (66.5)	62 (55.9)	25 (47.2)	
II	50 (9.7)	28 (8.0)	12 (10.8)	10 (18.9)	
III	119 (23.1)	78 (22.2)	26 (23.4)	15 (28.3)	
IV	26 (5.0)	12 (3.4)	11 (9.9)	3 (5.7)	

*Note*: *, these variables were presented as median (interquartile range) and compared by using Mann–Whitney test.

Abbreviations: MSI, microsatellite instability; N/A, not available; NSMP, no specific molecular profile.

**FIGURE 1:**
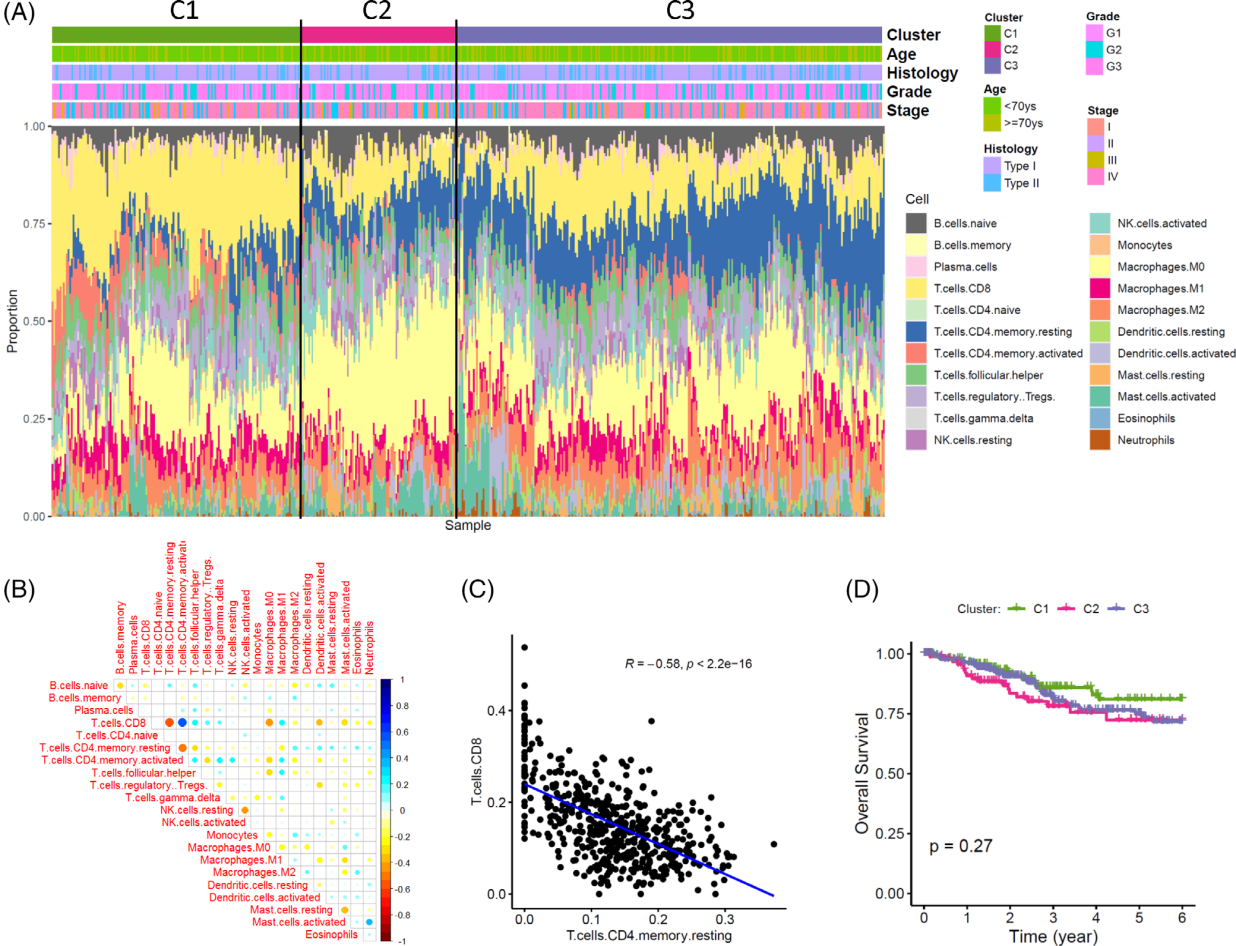
Overall immune landscape of endometrial cancer and its clinical relevance. (A) The relative fractions of 22 immune cells estimated by CIBERSORTx are presented with different colors. Samples are grouped into three subtypes (C1, C2, and C3) by unsupervised hierarchical clustering analysis using profiles of all immune cells. The distribution of clinicopathological variables among subtypes are depicted and showed no statistical significance (all *p* > .05). (B) Inter‐cell correlations of the 22 immune cells were calculated by Spearman correlation. The correlation coefficients *rho* was present in the matrix plot by different colors, with red for negative correlation and blue for positive correlation. (C) A representable dot plot for the negative correlation between CD8+ T cells and resting memory CD4+ T cells. (D) Survival analysis for the overall immune landscape by using Kaplan–Meier curves and log‐rank test.

The relative fractions of 22 immune cells in 516 tumor samples were successfully estimated by the CIBERSORTx algorithm (*p* < .01). The abundance of 22 subtypes of immune cells across 516 endometrial cancers were presented in Figure 1A. As the major cytotoxic cells in the tumor microenvironment, the fractions of CD8+ T cells were negatively associated with those of resting memory CD4+ T cells, M0 macrophages and activated dendritic cells (Figure 1B). On the other hand, the infiltrating levels of CD8+ T cells were positively correlated with those of activated memory CD4+ T cells, follicular helper T cells and M1 macrophages (Figure 1B). Particularly, the negative correlation between the levels of CD8+ T cells and resting memory CD4+ T cells was presented in Figure 1C (*ρ* = −0.58, *p* < .001).

Three major immune subtypes were identified by the 22 immune cells using unsupervised hierarchical clustering algorithm (termed as C1, C2, and C3). The C1 subtype was characterized as high‐infiltrated with CD8+ T cells, while increased levels of resting memory CD4+ T cells were observed in the C3 subtype. In subtype C2, both CD8+ T and resting CD4+ T levels were decreased, while the fractions of M0 macrophage were increased. However, there was no significant correlation between the three immune subtypes and other clinicopathological variables, including age, histology type, tumor grade, and stage. Although the C2 subtype seemed to have worse prognosis than the C1 subtype, the difference in prognosis was not significant (*p* > .05). These results indicated that the overall immune landscapes classified by all 22 immune cells may be complex and limited in predicting outcome.

### 3.2 Development of the TICS for endometrial cancer

**FIGURE 2:**
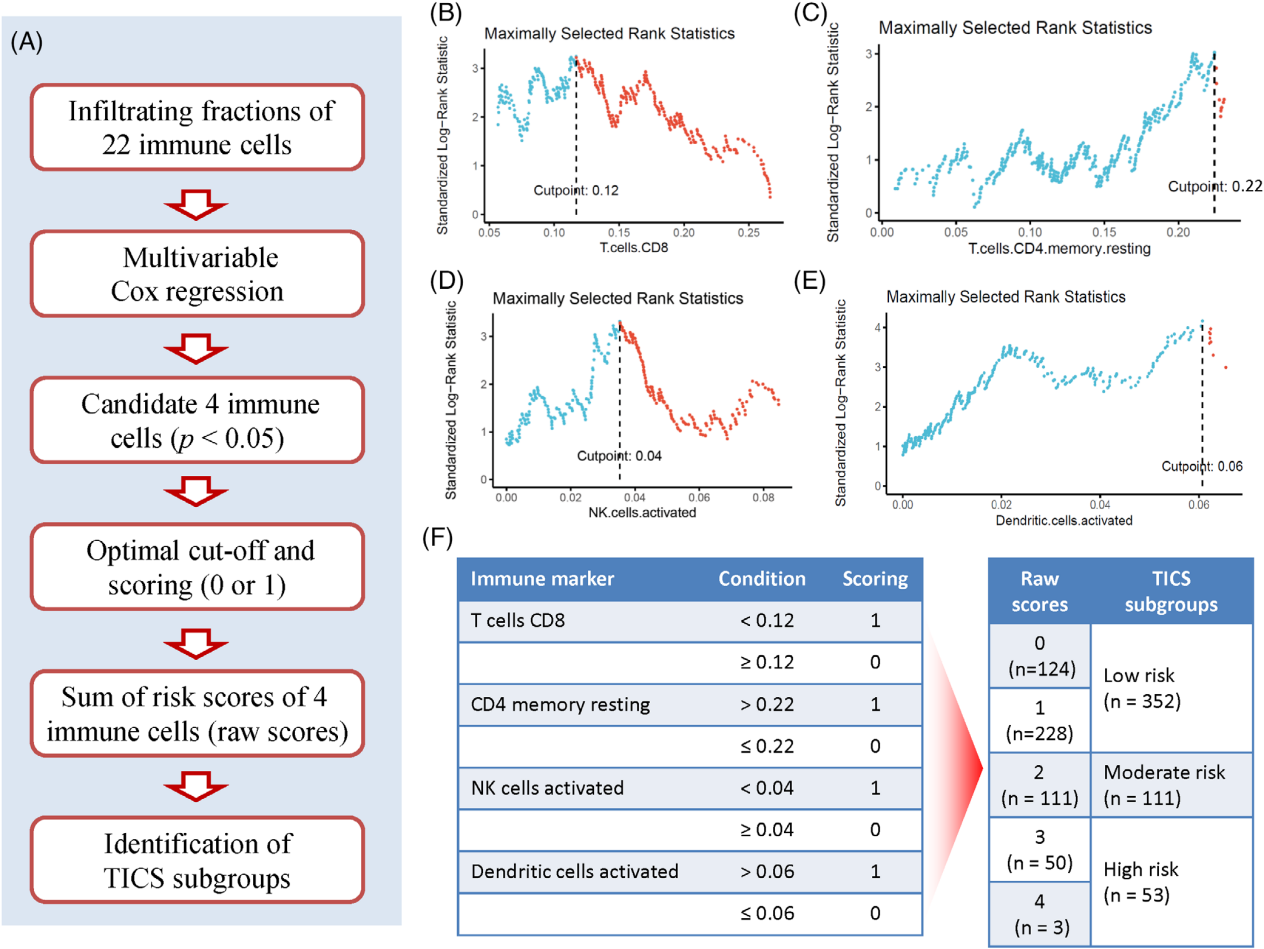
Construction of the Tumor‐infiltrating Immune Cell Score (TICS) for endometrial cancer. (A) The workflow of the process of construction of TICS. (B–E) Identification of the optimal cut‐offs for four immune cells in predicting OS. For each cell, the optimal cut‐off was defined as the point which yields the maximally selected rank statistics. (F) Scoring and combination of four immune cells. Patients would get 1 point if they were classified as high‐risk by the optimal cutoff of each immune cells (left panel). The raw prognostic immune cell score was defined as the sum of risk points of four immune cells. The groups with similar outcome as showed in Supplementary Figure S1 were merged into three subgroups of patients with low‐, moderate‐ and high‐risk (right panel).

The workflow diagram for developing TICS was illustrated in Figure 2A. The prognostic value of the 22 immune cells was estimated by using multivariable Cox regression model (Supplementary Table S1). After adjusting for age, tumor stage and grade, higher percentage of CD8+ T cells and activated NK cells significantly correlated with better survival (HR: 0.05, *p* = .025; and HR: < 0.01, *p* = .026), whereas higher fractions of resting memory CD4+ T cells and activated dendritic cells were associated with unfavorable prognosis (HR: 31.43, *p* = .015; and HR: 975.08, *p* = .008, respectively).

All patients were divided into two groups based on the optimal value of each cell population in predicting OS (Figure 2B–E). Patients received 1 point if they had lower fractions of CD8T cells (<0.12), higher resting memory CD4T cells (>0.22), lower activated NK cells (<0.04), or higher activated DCs (>0.06) (Figure 2F). The raw immune score (from 0 to 4) was defined as the sum of points obtained from 4 immune cells. Kaplan–Meier curves demonstrated the raw immune score was a predictor for OS rate, which was gradually decreased along with increased scores from 0 to 4 (Supplementary Figure S1, *p* < .001). However, the difference in OS between the raw immune score 1 and 0 was not statistically significant (HR = 1.38, *p* = .406). Similarly, the difference in OS between the raw score 4 and 3 was not statistically significant (HR = 1.38, *p* = .676). Accordingly, the tumor‐infiltrating immune cell score (TICS) was defined as follows: score of 0 or 1, low‐risk group; score 2, moderate‐risk group; and score of 3 or 4, high‐risk group.

### 3.3 Association of the TICS with clinicopathological and biological characteristics

**FIGURE 3:**
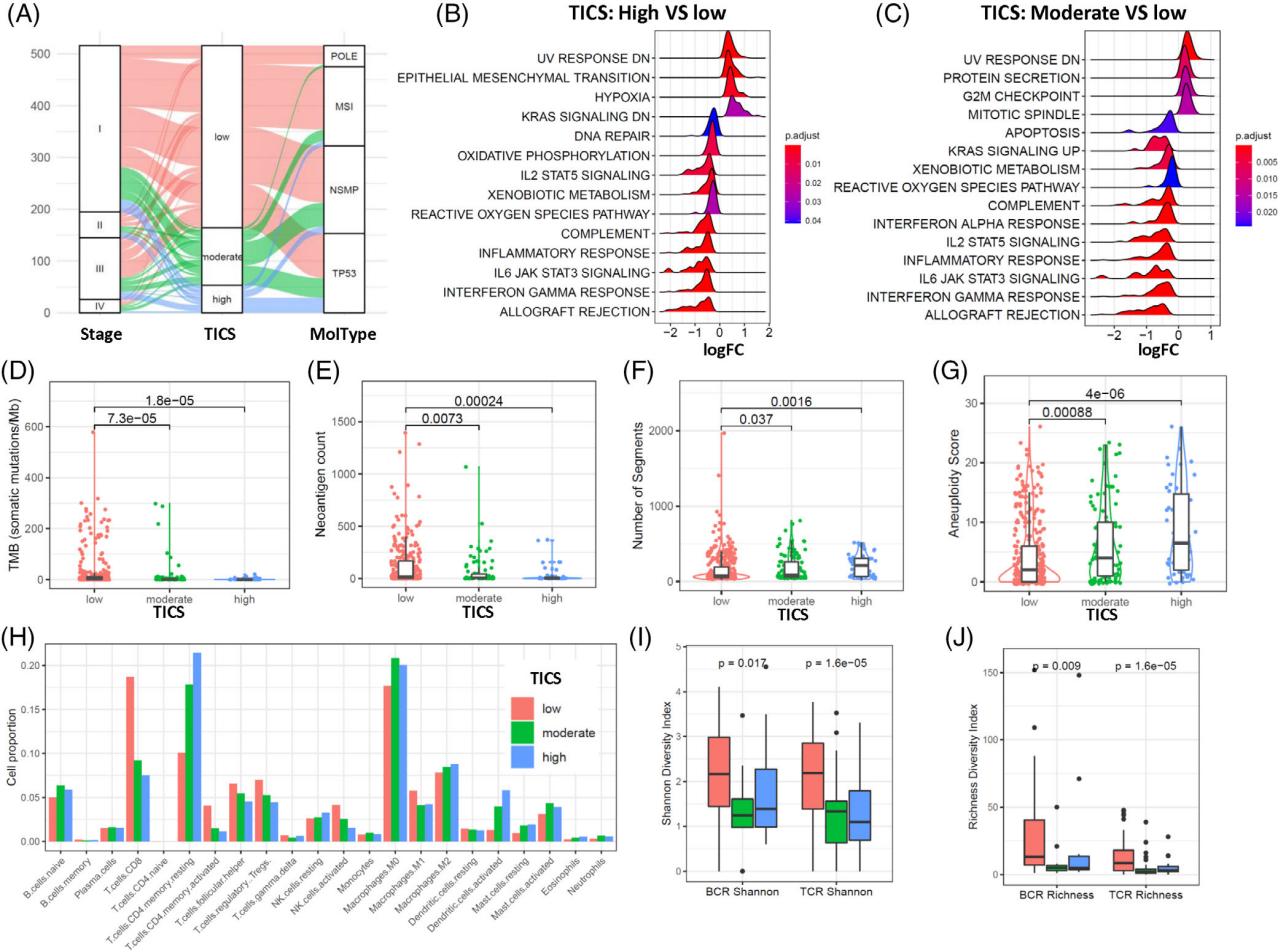
Clinicopathological and biological relevance of Tumor‐infiltrating Immune Cell Score (TICS). (A) Distribution of patients among TICS, stage and molecular type. Alluvial plot shows the distribution of patients as presented by curves with different colors assigned by TICS subgroups. (B, C) Gene Set Enrichment Analysis of the TICS subgroups. The 50 hallmark gene sets from the MSigDB were analyzed for the high‐risk (B) and moderate‐risk (C) subgroups as compared to the low‐risk subgroup. The ridge plot shows the distribution of log‐transformed fold change (FC) of each significant gene set (adjusted *p*‐value <.05). (D–G) Genetic alterations of the TICS subgroups. Box plots show the differences of tumor mutation burden (D), neoantigen count (E), DNA segment number (F) and aneuploidy score (G) among the TICS subgroups. The *p* value was calculated using Mann–Whitney test. (H) Bar plot of fractions of 22 immune cells among the TICS subgroups. (I, J) Immune diversity of the TICS subgroups. Box plots show the differences of T‐cell repertoire (TCR) and B‐cell repertoire (BCR) among the TICS subgroups. The Shannon diversity index (I) and Richness diversity index (J) are presented respectively. The *p*‐value were calculated using Mann–Whitney test.

There are no significant differences in the distribution of age, height, weight, menopause status, histologic type and tumor grade among the three groups (Table 1). Patients in the low‐risk group had higher fractions of MSI subtype and POLE subtype (34.4% vs. 19.8% vs. 18.9%; and 10.2% vs. 4.5% vs. 0%, respectively), but lower fraction of TP53 subtype (24.4% vs. 34.2% vs. 54.7%, *p* < .001) than patients in the moderate‐ and high‐risk groups. Patients in the low‐risk group had higher stage I percentages (66.5% vs. 55.9% vs. 47.2%) but lower stage II percentages than patients in the moderate‐ and high‐risk groups (8.0% vs. 10.8% vs. 18.9%, *p* = .008). Specially, the distribution of patients in the TICS subgroups, TNM stage and molecular subtypes was presented in Figure 3A.

GSEA showed that epithelial mesenchymal transition, hypoxia, Kras signaling DN signatures were significantly enriched in patients with high‐risk group than in patients with low‐risk group (Figure 3A). The patients with moderate‐risk group showed high expression of gene signatures in mitotic spindle, G2M checkpoint and protein secretion (Figure 3B). On the other hand, the patients with low‐risk group were characterized as increased expression various immune response features, such as interferon gamma response, IL6/JAK/STAT3 signaling, IL2/STAT5 signaling and inflammatory response (Figure 3A,B).

Considering the genome alterations, patients in the low‐risk group had significantly higher tumor mutation burden (TMB) score and more neoantigen counts than those in the moderate‐ or high‐risk group (Figure 3D,E). There was an incremental trend of copy number variation (CNVs) segment number in low‐, moderate‐ and high‐risk groups along with a parallel increase in aneuploidy score (Figure 3F,G).

Further analysis confirmed that the infiltrating levels of CD8T cells and activated NK cells were decreased in the low‐, moderate‐ and high‐risk groups, along with an increasing trend of resting memory CD4 T cells and activated DCs (Figure 3H). The infiltrating levels of other cell types showed no significant differences among the three groups. Meanwhile, the BCR and TCR diversities were significantly higher in the low‐risk group than those in moderate‐ and high‐risk groups (Figure 3I,J).

### 3.4 Survival analysis of the TICS in patients with endometrial cancer

**FIGURE 4:**
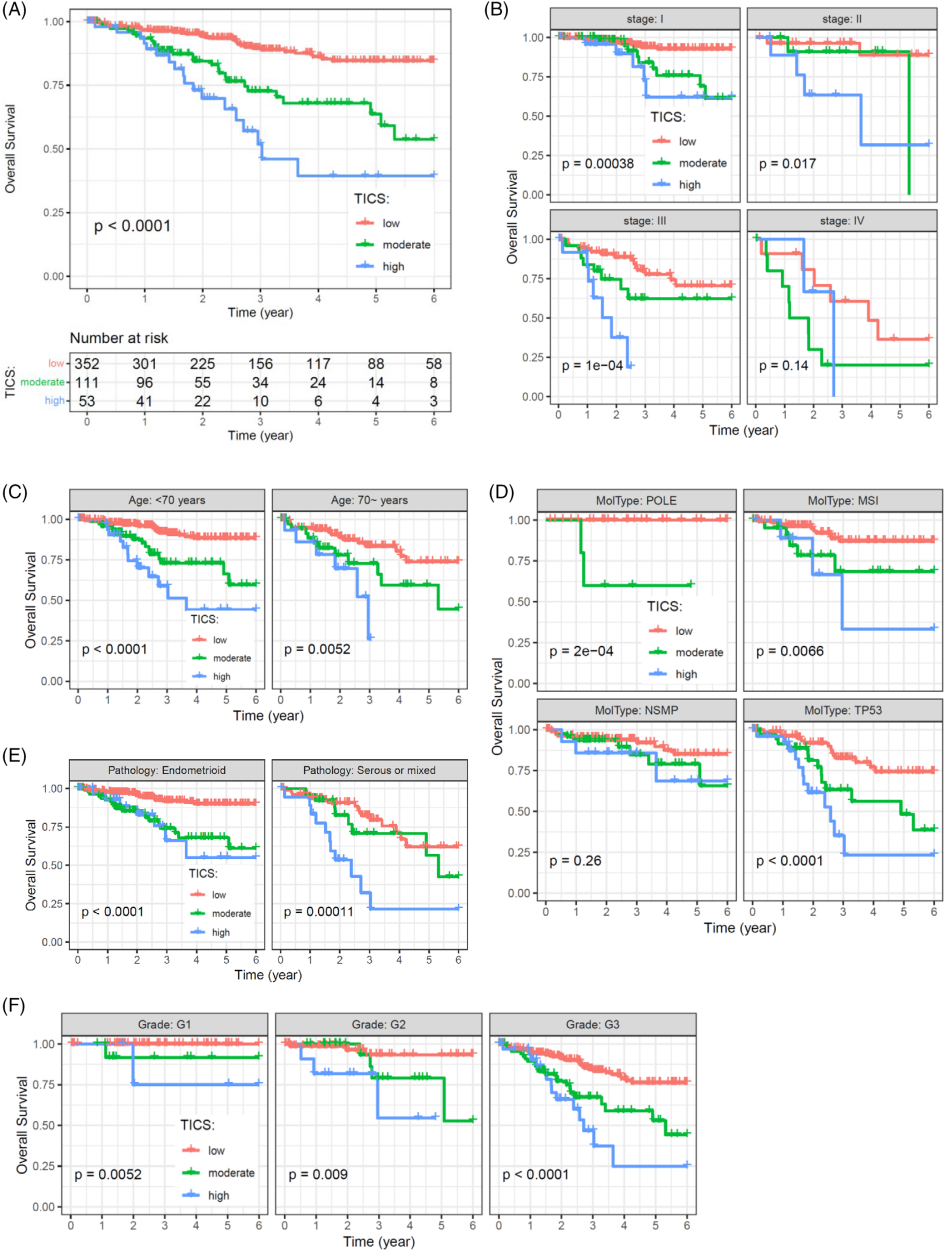
Survival analysis for OS in patients with EC. (A) Overall survival of the TICS subgroups by using the entire cohort. (B–F) Survival analysis for OS as stratified by the tumor stage (B), patient age (C), molecular classification (D), histology type (E), and tumor grade (F). Kaplan–Meier curves are used to compare the OS rates of different TICS subgroups, and *p*‐values were calculated by the log‐rank test.

Kaplan–Meier curves showed that the three groups defined by TICS were significantly associated with different OS rate (Figure 4A *p*<.001). The 5‐year OS for patients in the low‐, moderate‐ and high‐risk groups were 84.6% (95% CI, 79.6%–89.8%), 63.7% (95% CI, 51.5%–78.7%), and 39.5% (95% CI, 23.3%–66.8%), respectively.

The TICS significantly predicted OS in patients with stages I, II, and III diseases, respectively (Figure 4B, all *p* < .05). However, the difference in OS was not statistically significant in the stage IV subgroup, which may be attributed to the relatively small number of patients. The difference in OS among TICS subgroups was statistically significant after stratifying by patient age (Figure 4C, all *p* < .01). When considering molecular subtypes, TICS was a significant predictor for OS in the POLE, MSI, and TP53 subtypes (Figure 4D, all *p* < .01), but not in the NSMP subtype. Furthermore, we found that TICS could predict OS in both endometrioid histology and serous or mixed tumors, based on pathological classification (Figure 4E, all *p* < .001). When stratifying by tumor grade, difference in OS among TICS subgroups remained statistically significant (Figure 4F, all *p* < .01).

**FIGURE 5:**
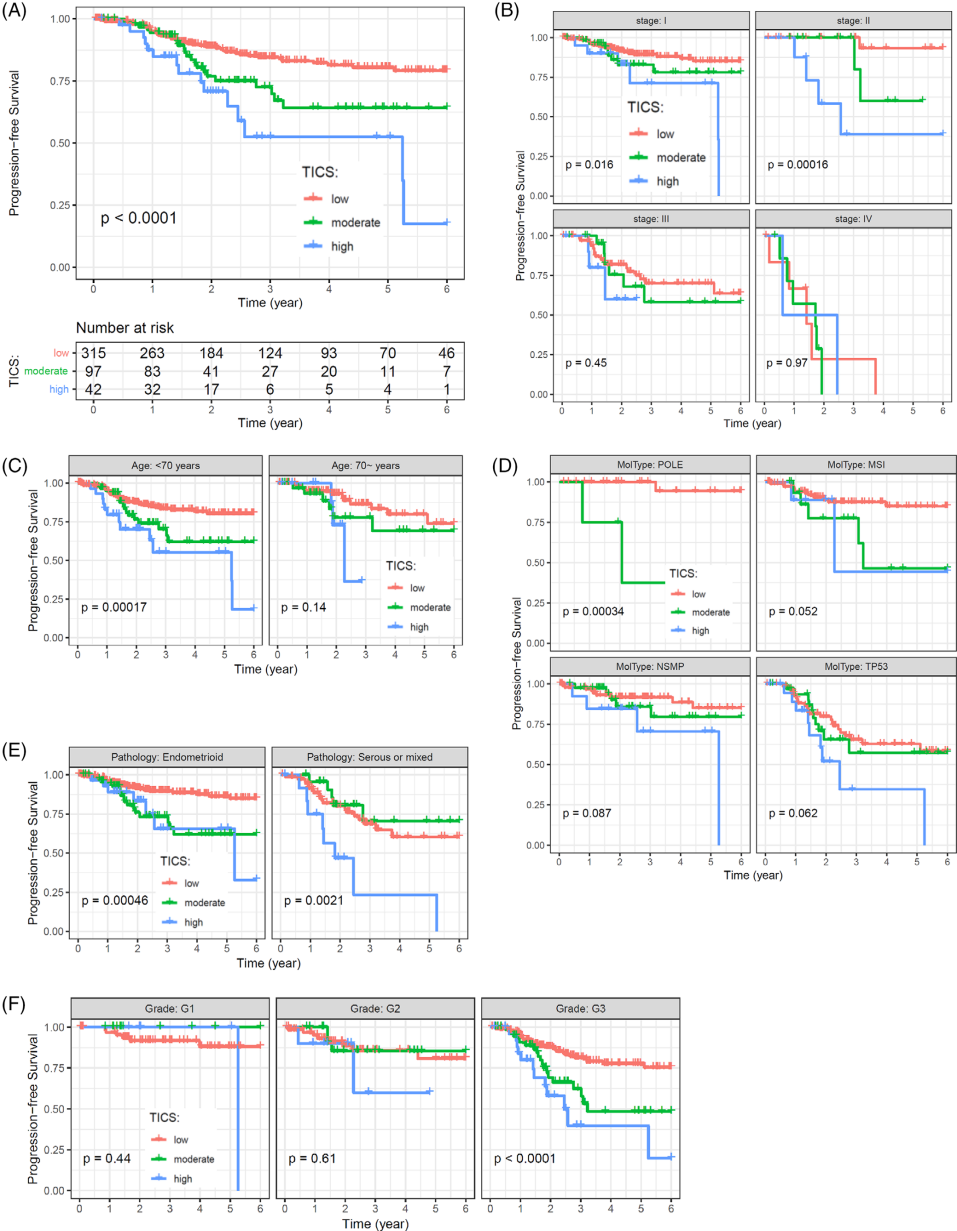
Survival analysis for PFS in patients with EC. (A) Progression‐free survival of the Tumor‐infiltrating Immune Cell Score (TICS) subgroups by using the entire cohort. (B–F) Survival analysis for PFS as stratified by the tumor stage (B), patient age (C), molecular classification (D), histology type (E), and tumor grade (F). Kaplan–Meier curves are used to compare the PFS rates of different TICS subgroups, and *p*‐values were calculated by the log‐rank test.

The prognostic value of the TICS was validated for PFS. Kaplan–Meier survival curve indicated that the TICS subgroups were significantly associated with PFS rate (Figure 5A *p* <.001). Meanwhile, the difference in PFS remains significant in patients with Stage I and II diseases, age of <70 years, different molecular subtypes, different pathology subtypes, and grade 3 tumors (Figure 5B–F). These results indicate that TICS was an independent prognostic factor for OS and PFS, irrespective of clinicopathological factors or molecular classification.

### 3.5 The prognostic value of TICS is independent of chemotherapy and radiotherapy

**FIGURE 6:**
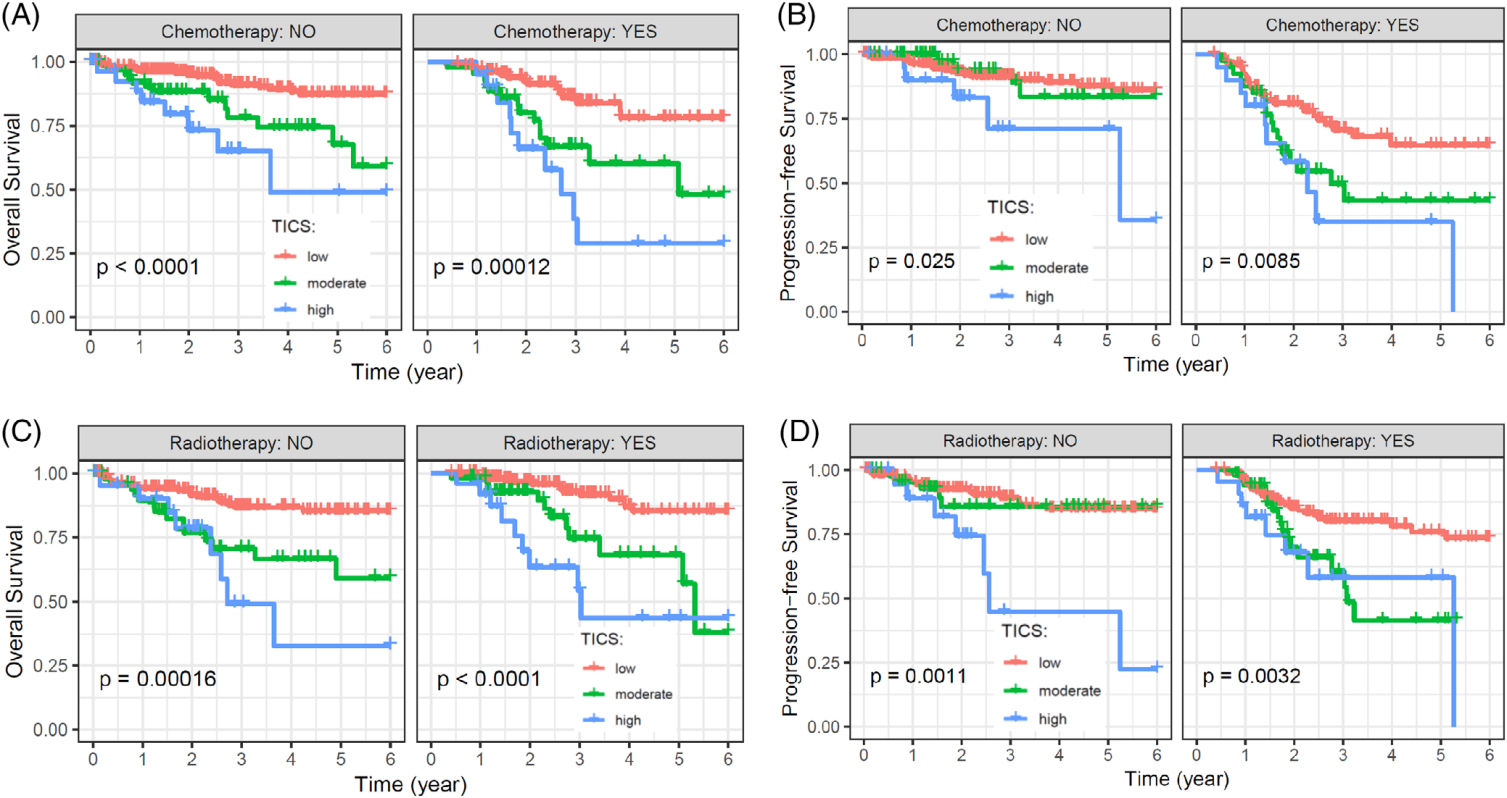
Survival analysis for overall survival (OS) and progression‐free survival (PFS) stratified by different treatment. (A), Survival analysis for OS in patients receiving chemotherapy or not. (B), Survival analysis for PFS in patients receiving chemotherapy or not. (C), Survival analysis for OS in patients receiving radiotherapy or not. (D), Survival analysis for PFS in patients receiving radiotherapy or not. Kaplan–Meier curves are used to compare the OS or PFS rates of different TICS subgroups, and *p*‐values were calculated by the log‐rank test.

We also investigated whether the prognostic value of TICS is influenced by treatment response. For the TCGA cohort, 170 and 249 patients received chemotherapy and radiotherapy after surgery, respectively. The low‐, moderate‐ and high‐risk subgroups of TICS were associated with OS in patients receiving chemotherapy and in those without chemotherapy (Figure 6A, both *p* < .001). Similar results were observed in the analysis for PFS (Figure 6B, both *p* < .001). According to radiotherapy, the TICS subgroups significantly predict OS and PFS, irrespective the patients receive radiotherapy or not (Figure 6C,D).

### 3.6 The TICS improved the prognostic ability of traditional clinicopathologic factors

In multivariable Cox analysis, three clinicopathologic factors including tumor stage, grade and age were found to be significant in predicting OS. An integrated model was developed by adding the TICS to the clinicopathologic risk factors (Table 2, all *p*‐values <.05). The C‐index for the integrated OS model was 0.80 (95% CI, 0.75–0.85) and the bias corrected C‐index by use of bootstrap validation was 0.78 (95% CI, 0.75–0.82). The improvement in C‐index of the integrated model was significant as compared with that of the clinicopathological model or the tumor stage alone (Table 3, all *p* < .05).

**TABLE 2:** Multivariable Cox regression analysis for Nomogram model.

Variable	Levels	HR (95% CI)	*p*‐Value
Age			
	<70 years	1 (reference)	
	≥70 years	2.07 (1.31–3.3)	.002
Stage			
	I	1 (reference)	
	II	1.23 (0.55–2.75)	.614
	III	3.63 (2.09–6.31)	<.001
	IV	6.52 (3.38–12.6)	<.001
Grade			
	G1	1 (reference)	
	G2	3.62 (0.79–16.62)	.098
	G3	6.15 (1.47–25.69)	.013
TICS			
	Low	1 (reference)	
	Moderate	2.85 (1.7–4.77)	<.001
	High	5.49 (3.02–9.99)	<.001
C‐index original: 0.80			
Bootstrap C‐index: 0.78			

Abbreviation: CI, confidence interval.

**TABLE 3:** C‐index analysis for TICS, pTNM and Nomogram model.

Predictors	C‐index (95% CI)	*p*‐Value*
TICS	0.66 (0.6–0.72)	<.001
pTNM	0.72 (0.66–0.77)	<.001
Clinicopathological model	0.77 (0.71–0.82)	.003
Nomogram model	0.80 (0.75–0.85)	–

*Note*: *the *p*‐values represent the comparison with the Nomogram model; TICS, tumor‐infiltrating immune cell score; CI, confidence interval; clinicopathological model, this model was conducted by age, grade and pTNM stage; Nomogram model, this model was conducted by TICS, age, grade and pTNM stage.

**FIGURE 7:**
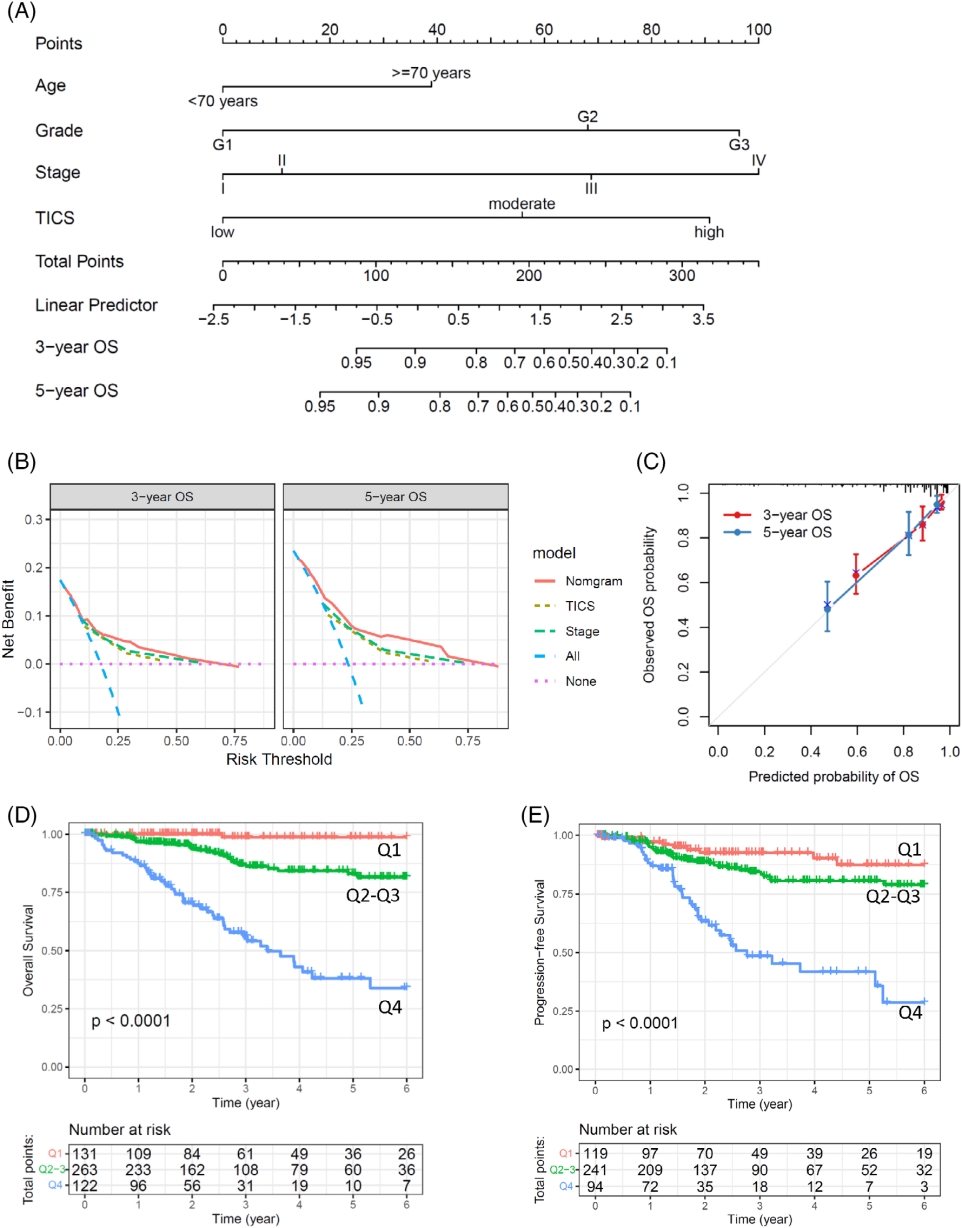
Survival nomogram for endometrial carcinoma (EC) patients. (A), The nomogram predicting for OS of EC patients. For each individual patient, the corresponding value is located on each variable axis, and a line is drawn upward to determine the number of points received for each variable value. Total Points axis represent the sum of these numbers, and two lines are drawn downward to the 3‐ or 5‐year survival axes. (B) The calibration curves of the nomogram are present for predicting patient survival at 3 years and 5 years. (C) Decision curves of different predictors are present for predicting patient survival at 3 years and 5 years. (D) Kaplan–Meier curves show the OS rates of three subgroups as defined by the nomogram predictor. (E) Kaplan–Meier curves show the PFS rates of three subgroups as defined by the nomogram predictor. The *p*‐values were calculated by the log‐rank test.

For easy‐clinic usage, a nomogram was constructed to estimate the predicted probability of 3‐year or 5‐year OS in the form of graphic depiction (Figure 7A). The points are assigned with the effect factors of TICS, tumor stage, tumor grade and age. For example, a patient with moderate TICS, stage III, grade G3, and 73 years old at diagnosed would have a total of 259.9 points and an estimated 3‐year and 5‐year OS of 29.8% and 14.4%, respectively. Calibration curves showed that the modeled 3‐year or 5‐year estimates of OS closely approximated the actual estimates in the training cohort (Figure 7B). Meanwhile, the decision curve showed that the nomogram achieved better benefit than TICS or TNM stage alone in predicting 3‐year and 5‐year OS, respectively (Figure 7C).

Kaplan–Meier curves showed significant differences in overall survival among the three groups defined by the model (Figure 7D *p*<.001). Three‐year OS rate for patients in the lower, middle, and upper groups were 98.6% (95% CI, 95.8%–100.0%), 82.9% (95% CI, 76.9%–89.3%), and 38.1% (95% CI, 27.4%–53.2%), respectively. Meanwhile, the three groups also revealed significant differences in PFS (Figure 7E).

## 4 DISCUSSION

In this study, we have developed a scoring system named Tumor‐infiltrating Immune Cell Score (TICS) to predict overall survival in patients with ECs. The TICS comprises CD8T cells, resting memory CD4 T cells, activated NK cells and activated DCs, and has demonstrated independent prognostic ability in predicting OS and RFS. The low‐risk subgroup identified by TICS was associated with MSI or POLE subtype, higher TMB score, and activation of various immune response signaling. On the other hand, the high‐risk subgroup was significantly correlated with higher fraction of TP53 subtype, activation of EMT, hypoxia and KRAS signaling. We constructed a nomogram predictor by integrating TICS, age, tumor stage and tumor grade, which demonstrated a significantly improved prognostic ability compared with the clinicopathological model alone.

Tumor microenvironment is so complex that the prognostic role of tumor infiltrating immune cells remains to be controversial in patients with EC. Previous studies have shown that high numbers of CD8+ T cells^7^, ^8^ and CD57 + NK cells^23^, ^24^ in tumor tissues were associated with improved prognosis and in patients with EC. On the other hand, the tumor infiltrating immune suppressive cells such as FoxP3+ Tregs and CD68+ macrophages were correlated with inferior outcome.^12^ However, a recent study reported that the immune classification of EC based on tumor infiltrating CD8+ T cells, CD4+ T cells, Tregs, B cells, and plasma cells could not predict patient survival.^15^ In this study, we identified three tumor subtypes based on the relative fraction of 22 immune cells representing the overall immune landscape of EC. However, the three immune subtypes were not significantly correlated with overall survival, indicating that the classification based on overall unselected immune cells may provide less prognostic information.

To address this issue, we established a TICS which comprises lower levels of CD8 T cells and NK cells, higher levels of resting memory CD4 T cells and activated DCs. It has been shown that a high density of cytotoxic immune cells, such as CD8 T cells and NK cells, in the tumor microenvironment is correlated with improved outcome in patients with EC.^7^, ^8^, ^24^ On the other hand, the presence of resting memory CD4T cells indicates impaired immune response, and is associated with unfavorable prognosis in cases of melanoma, urothelial cancer, thyroid carcinoma, gastric cancer and lung adenocarcinoma.^25^, ^26^, ^27^, ^28^ While DCs are thought to present tumor‐associated antigens to naive T cells and contribute to an anti‐tumor role, our findings indicated that high levels of activated DCs within the tumor were inversely correlated with densities of CD8 T cells, and predicted worse outcome for patients with EC. Jensen et al.^29^ reported that higher levels of intratumoral plasmacytoid dendritic cells (CD123+) were ultimately correlated with worse survival, indicating that DCs play a dual role in tumor progression. These results suggest that an immunosuppressive microenvironment and lower infiltration of cytotoxic cells were effective predictors for outcome of EC patients.

For the past decade, the molecular classification based on genomic mutations and DNA copy number variations has improved the risk stratification and clinical management of EC.^30^, ^31^ The POLE ultramutated group carries somatic mutations in the POLE gene and has an excellent prognosis without recurrence. The MSI subgroup is characterized by defects in the mismatch repair system, which was considered to benefit from immune checkpoint inhibitors. TP53‐mutant subtype is characterized by P53 abnormalities and high number of somatic alterations such as high copy number variation. This subtype has the worst prognosis. The NSMP subtype presents low genomic alterations and low DNA copy number, but the ER/PR were highly expressed. Meanwhile, other prognosis relevant molecular alterations are recently identified, such as abnormal expression of L1CAM and ARID1a, mutations in PPP2R1a, FBXW7, CTNNB1, and PI3K/AKT.^31^ These results suggest that it is necessary to perform more detailed classification especially in high‐risk patients. Except for NSMP subtype, the POLE ultramutated, MSI and TP53‐mutant subtypes carry high levels of genomic alterations which offer the possibility to stimulate immune response. In this study, we found that the TICS independently predict patient survival in POLE ultramutated, MSI and TP53‐mutant subtypes. These results suggest that the status of immune microenvironment may synergistically influence the prognosis and clinical management of EC patients, and should be considered together with the molecular classification.

The tumor immune microenvironment is affected by genome alterations in cancer cells. Microsatellite instability and *POLE* ultramutation are common molecular subtypes of ECs, which are characterized by genome‐wide DNA instability and mutations.^4^ The accumulation of numerous genetic mutations accumulated by cancer cells promotes the generation of tumor neoantigens, thereby stimulating anti‐tumor immune responses.^32^, ^33^ In our study, tumors with MSI and POLE mutation were predominantly enriched in the low‐risk subgroup assigned by TICS. Meanwhile, the tumor mutation burden and neoantigen counts were significantly higher in the low‐risk subgroup. These genetic alterations may contribute to the activation of immune response, as evidenced by significantly higher TCR and BCR diversity. Conversely, the DNA copy number variation and aneuploidy score were significantly higher in the moderate‐ and high‐risk subgroups compared to the low‐risk subgroup. Previous studies have reported that DNA copy number alteration and aneuploidy are associated with aggressive phenotype and poor survival in ECs.^34^, ^35^ Furthermore, CNV levels were negatively correlated with immune cell infiltration in tumor tissues.^36^, ^37^ These results suggest that, unlike DNA mutation, changes in DNA quantity may have little effect on immune activation and lead to unfavorable outcome in patients with ECs.

The TP53‐mutant subtype has the worst prognosis and the most common tumors are serous and mixed carcinomas.^5^, ^21^ However, the heterogeneity of immune characteristics within the TP53‐mutant subtype remains unclear. Wang et al. analyzed the expression pattern of gene signatures such as T‐effector, IFN‐γ, anti‐PD‐1 resistance and CD8+ T cell exhaustion in four molecular subtypes.^38^ The results showed that the immune microenvironment of TP53‐mutant subtype exhibited great heterogeneity, which was associated with prognosis of patients. Consistently, our results also showed that the immune cell score of tumors was significantly correlated with prognosis in the TP53‐mutant subtype. The patients who were classified as low‐risk by the TICS in the TP53 subtype had a similar survival rate with those in the NSMP subtype. On the other hand, the patients classified as high‐risk of TICS in the MSI subtype would have worse outcome. These results suggest that TICS could be a supplement to EC molecular subtyping to improve the accuracy of prognosis and may guide clinical practice such as immunotherapy.

In our results, we found that TICS subgroups were significantly associated with traditional prognostic factors such as histological classification, tumor stage and tumor grade. Nevertheless, the prognostic value of TICS was independent of these factors, as demonstrated by stratification analysis. The prognostic value of TICS remained significant in each subgroup stratified by age, morphological type, tumor stage, and tumor grade. Furthermore, multivariable Cox regression analysis confirmed that TICS is an independent predictor of overall survival. The integrated model that incorporated TICS and clinicopathological factors demonstrated significant improvement in prognostic ability compared to the model constructed with clinicopathological factors alone. These results indicate that TICS may be a possible supplement to traditional factors used to predict the prognosis of patients with EC.

Although our research has established a prognostic model for EC from the perspective of multiple tumor‐infiltrating immune cells, there are still several limitations of this study. We only analyzed one cohort from the TCGA database, and only internal cross‐validation was performed. Additionally, the immune cell compositions were calculated by CIBERSORTx based on gene expression data. The expression levels and prognostic roles of molecules at the functional level, such as proteins, remain unclear. It is recommended to use novel quantitative techniques, such as multiplex immunohistochemistry, to quantify immune cells and to verify the signature in external independent EC cohorts.

## 5 CONCLUSIONS

In summary, our study suggests that specific immune cell populations, rather than the overall immune landscape, are associated with the prognosis of patients with EC. The TICS, which comprises CD8 T cells, resting memory CD4 T cells, activated NK and activated DCs, represents an effective prognostic factor for EC patients and could be a useful supplement to existing clinicopathological factors and molecular classification. Our findings also highlight the intricate interplay among genomic alterations, immune cell infiltration, and the tumor microenvironment in EC, which may have significant implications for personalized cancer therapy.

## AUTHOR CONTRIBUTIONS

**Liping Zhang:** Data curation (equal); formal analysis (equal); investigation (equal); writing – original draft (equal). **Qiaoying Zhu:** Data curation (equal); investigation (equal); writing – original draft (equal). **Qi Zhao:** Investigation (equal); methodology (equal). **Xueping Lin:** Methodology (equal); writing – review and editing (equal). **Hui Song:** Methodology (equal); writing – review and editing (equal). **Hong Liu:** Methodology (equal); writing – review and editing (equal). **Guiquan Zhu:** Supervision (equal); writing – review and editing (equal). **Shun Lu:** Supervision (equal); writing – review and editing (equal). **Bangrong Cao:** Conceptualization (lead); formal analysis (equal); funding acquisition (lead); project administration (lead); writing – review and editing (lead).

## FUNDING INFORMATION

This work was supported by grants from the National Natural Science Foundation of China (Grant No. 82272818), Natural Science Foundation of Sichuan Province (Grant No. 22NSFSC0850) and the Cancer Medical and Engineering Research Foundation of University of Electronic Science and technology (Grant No.ZYGX2021YGCX009).

## CONFLICT OF INTEREST STATEMENT

The authors have stated explicitly that there are no conflicts of interest in connection with this article.

## ETHICS STATEMENT

This work does not include human participants or animals performed by any of the authors, therefore no ethic approval or consent is required.

## Supporting information


**Data S1:** Supporting Information.

## Data Availability

The initial gene expression data and clinical information were publicly available in The Cancer Genome Atlas. The processed data that support the findings of this study are available on request from the corresponding author.
