# Supplementary material for: Tumor‐infiltrating immune cell score as an independent prognostic predictor for endometrial carcinoma: Insights from a comprehensive analysis of the immune landscape
Source: Cancer Rep (Hoboken). 2023 Nov 28;7(1):e1939. doi: 10.1002/cnr2.1939 (PMC10809205; doi:10.1002/cnr2.1939)
Supplement: Supplementary file 1 — Data S1: Supporting Information. [file CNR2-7-e1939-s001.pdf]

Supplementary **Table S1**. Cox regression analysis for 22 immune cells

Features	Hazard Ratio	lower.95%CI	upper.95%CI	*P* value
B.cells.naive	12.59	0.01	12761.87	0.473
B.cells.memory	0.00	0.00	4.48e10	0.451
Plasma.cells	1.62	0.00	59488.54	0.928
T.cells.CD8	0.05	0.00	0.68	0.025
T.cells.CD4.naive	0.00	0.00	Inf	0.996
T.cells.CD4.memory.resting	31.43	1.95	507.23	0.015
T.cells.CD4.memory.activated	0.00	0.00	1.16	0.056
T.cells.follicular.helper	0.02	0.00	52.36	0.327
T.cells.regulatory..Tregs.	0.01	0.00	16.44	0.222
T.cells.gamma.delta	0.76	0.00	981573.74	0.970
NK.cells.resting	4.04	0.00	4630.51	0.698
NK.cells.activated	0.00	0.00	0.38	0.026
Monocytes	10142.77	0.00	1.05e12	0.327
Macrophages.M0	1.25	0.13	11.53	0.845
Macrophages.M1	4.20	0.00	8522.07	0.712
Macrophages.M2	117.68	0.76	18196.46	0.064
Dendritic.cells.resting	32.11	0.00	1.35e8	0.656
Dendritic.cells.activated	975.08	6.08	156297.00	0.008
Mast.cells.resting	2141.43	0.13	3.59e7	0.122
Mast.cells.activated	1.00	0.00	217.79	0.999
Eosinophils	5407.64	0.00	6.30e16	0.576
Neutrophils	0.00	0.00	4381370.34	0.467

Note: CI, confidence interval. The cox regression analysis for each immune cell was adjusted by tranditional prognostic factors including age, tumor stage and grade.

Supplementary **Figure S1**. Survival anlaysis of the raw scores by four immune cells

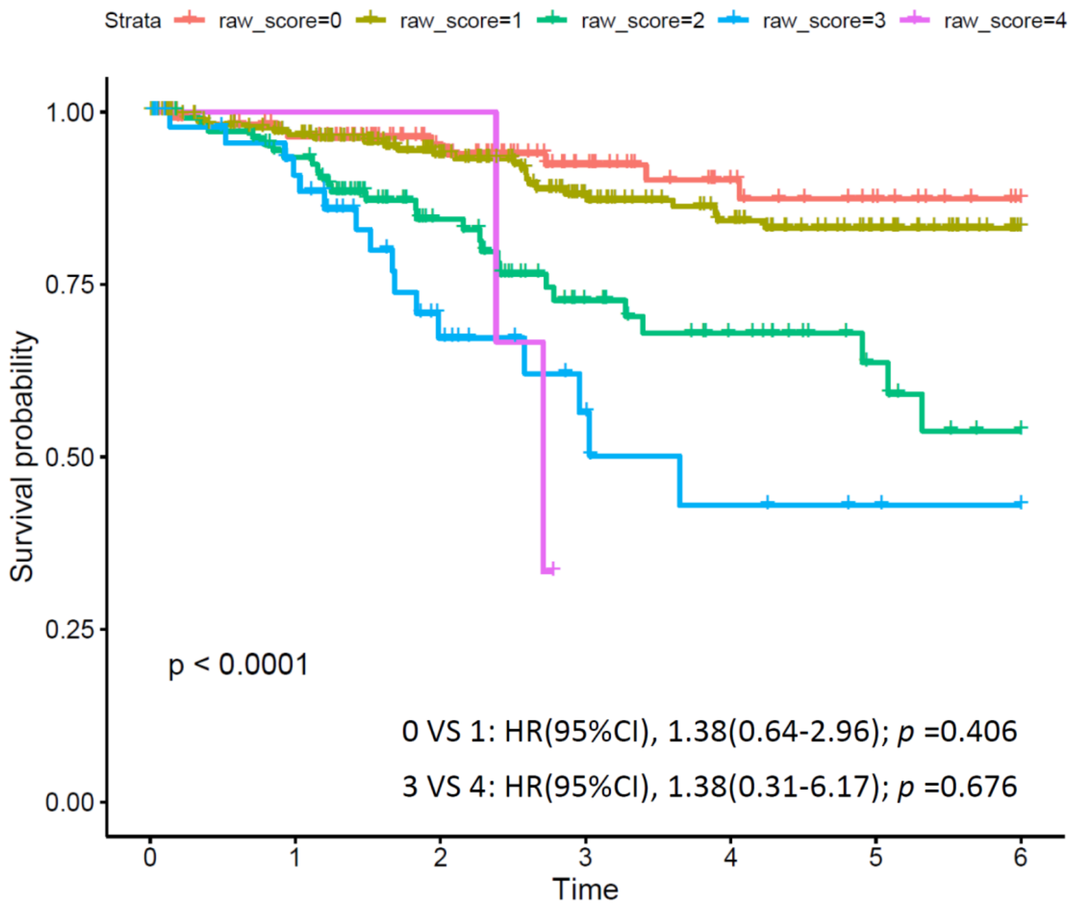

Figure S1. Survival anlaysis of the raw scores by four immune cells. Kaplan-Meier curves were used to compare the OS rates of different TICS subgroups, and *p* values were calculated by the log-rank test. Cox regression analysis was conducted to compare the difference between group 0 and group 1, and group 3 and group 4.
